# Effects of Cognitive Reappraisal on Subjective and Neural Reactivity to Angry Faces in Children with Social Anxiety Disorder, Clinical Controls with Mixed Anxiety Disorders and Healthy Children

**DOI:** 10.1007/s10578-021-01173-y

**Published:** 2021-04-24

**Authors:** Verena Keil, Brunna Tuschen-Caffier, Julian Schmitz

**Affiliations:** 1 Department of Clinical Psychology and Psychotherapy, University of Freiburg, Freiburg, Germany; 2 Department of Clinical Child and Adolescent Psychology, Leipzig University, Leipzig, Germany; 3 Leipzig Research Center for Early Child Development, Leipzig University, Leipzig, Germany

**Keywords:** Event-related potentials, Reappraisal, Gender, Emotional face processing, Children, Social anxiety disorder

## Abstract

Cognitive models of social anxiety suggest that social anxiety disorder (SAD) is characterized by both enhanced emotional reactivity and deficits in emotion regulation. Emotional reactivity to socially threatening children’s faces and their modulation through reappraisal were measured via subjective ratings and electrocortical responses in children (age 10–13) with SAD (*n* = 28), clinical controls with mixed anxiety disorders (*n* = 28), and healthy controls (*n* = 29). Children with SAD showed higher subjective reactivity to the images of angry children’s faces while all children reported reduced reactivity in their subjective ratings following reappraisal. Reduced electrocortical reactivity after reappraisal was only evident in older children and boys and was unrelated to anxiety. The present study indicates that cognitive reappraisal may be beneficial in reducing subjective reactivity in children with anxiety disorders, while neural effects of reappraisal may emerge at older ages.

## Introduction

Social anxiety disorder (SAD) is one of the most prevalent mental disorders in childhood [1, 2]. SAD is characterized by an intense fear of negative evaluation, and can result in a significant reduction in quality of life for children [3]. Despite SAD’s high prevalence in childhood, the factors supporting the maintenance of the disorder in children are poorly understood [4].

### Emotional Reactivity and Regulation in Childhood SAD

Cognitive models and diagnostic criteria suggest that SAD is maintained by heightened emotional reactivity to social stimuli as a result of biased cognitions, such as distorted appraisals of social situations (e.g. “I am stupid and others don’t like me”). In accordance with these theoretical assumptions, several studies of adult SAD populations have found processing biases and enhanced reactivity to socially threatening stimuli such as angry faces (see [5] for a review) as possible maintenance mechanisms. Another key maintenance mechanism may be maladaptive emotion regulation. Research suggests that *adults* with SAD use maladaptive emotion regulation strategies (e.g. suppression, avoidance) more frequently, while also showing deficits in adaptive emotion regulation strategies such as reappraisal [6]. Research on emotional reactivity and emotion regulation in *childhood SAD* is scarce. It has been found that children with mixed anxiety disorders (including SAD) show heightened negative subjective responses to threatening images as well as less frequent/less effective use of reappraisal compared to healthy controls [7–9].

When investigating emotional reactivity and regulation, research increasingly employs neural and physiological measures of reactivity and regulation in children. Complementing subjective assessment of emotion regulation in childhood research beyond subjective measures is important, since the validity of self-reports of children can be restricted by lower levels of cognitive-emotional development as well as lower self-awareness and may contain significant biases such as memory biases, report tendencies and social desirability [10, 11]. Furthermore, applying neural and physiological measures in childhood psychopathology can shed light on the intriguing question whether biological changes related to psychopathology are present at early stages of disorder development already or whether those changes develop later when disorders become more chronic [12].

Event related potentials (ERPs) have proven particularly useful in this regard, providing a time-sensitive, non-invasive method to elucidate neural correlates of the rapidly unfolding processes of emotion regulation [13, 14]. ERPs have been used to examine the dynamic interplay between bottom-up, stimulus-driven attention to emotional stimuli (i.e. emotional reactivity) and their top-down modulation via regulation strategies. Heightened emotional reactivity, as indexed by elevated ERP responses to emotional stimuli, has been reported in children via early ERP components (P100, N170, EPN) as well as later components such as the P3 and late positive potential (LPP; [15]). To date, however, only a handful of ERP studies have examined emotional reactivity specifically in children with SAD. No differences have been reported between children with SAD and healthy controls via early ERP reactivity in response to emotional faces [16–18]. However, a recent study found enhanced P100 and N170 in children with mixed anxiety disorders (including SAD; [19]). In late ERPs (LPP), by contrast, the findings are also mixed: some studies report increased LPPs in response to threatening faces in children with SAD [17, 18] and in mixed anxiety disorder groups (including SAD; [20]), while another study did not find amplified LPPs in response to angry faces in children with SAD [16].

The ERP responses generated when employing emotion regulation strategies, such as reappraisal, are mostly found in later stages of stimulus processing (i.e. during the P3/LPP time window). The LPP was reduced in healthy children when instructed to reappraise by interpreting threatening images as neutral [21, 22]. This LPP reduction has been thought to reflect successful emotion regulation via reappraisal. Reappraisal ability seems to improve continuously from childhood until early adulthood, likely due to prefrontal cortex maturation [23, 24]. Consequently, studies report linear relationships between enhanced reappraisal-induced LPP modulation and increasing age [22, 25]. Taken together, these results suggest that the LPP can be modulated by reappraisal, particularly in older children, but no study to date has investigated how these effects present in children with SAD.

### Limitations of Previous Research and the Present Study

While previous research has provided important information regarding emotional reactivity and regulation in childhood SAD, there are several notable limitations, which may also account for some inconsistencies in previous ERP studies on emotional reactivity in childhood anxiety. First, some studies investigated relatively small samples [17, 18] and may have been statistically underpowered to capture small or medium sized group differences in neural indices. Furthermore, due to the lack of clinical control groups, little is known about transdiagnostic or disorder-specific effects of emotion regulation processes [26]. The ongoing debate on the universal Research Domain Criteria approach (RDoC; [27]) has led researchers to speculate that different anxiety disorders might share more common biological, cognitive and behavioral maintaining processes than suggested by their distinct classification categories in the DSM-V [28]. For example, Lang et al. [29] found that the physiological reactivity profiles of different anxiety disorders were very similar when anxiety disorders with comparable disorder severity were examined. This calls particularly for the inclusion of clinical control groups (e.g. individuals with mixed anxiety disorders), which can provide more insight into the question if emotional and biological findings are specific for particular anxiety disorders such as SAD or relate more generally to the clinical anxiety spectrum.

Second, some studies investigated a broad age range [7, 8, 20], which may be problematic since emotional reactivity, emotion regulation and related neural correlates differ considerably between children and adolescents [30, 31]. Third, existing studies with children have investigated ERP reactivity using pictures of adult faces. Evidence suggests that children may process peer and adult emotional faces differently [32, 33]. When investigating emotional reactivity in response to social threat in childhood SAD, children’s faces may have higher salience and more ecological validity since age matched peers are their primary source of social evaluation [34]. Last, most studies on emotional reactivity and emotion regulation in children with anxiety disorders did not consider potential gender differences between boys and girls. This may be problematic, because previous research suggests male and female patients to differ both in terms of their emotional reactivity as well as their ability to regulate emotions including cognitive reappraisal [35, 36]. Hence, it is conceivable that patients with clinical anxiety do not generally differ from non-anxious groups but that more subtle differences exist specifically between either anxious and non-anxious females or anxious and non-anxious males, which would remain uncovered if gender differences were not taken into account.

The present study is the first to investigate the subjective and electrocortical reactivity to socially threatening children’s faces and their modulation through reappraisal in children with SAD. It is also the first study to compare the responses of children with SAD to healthy and clinical control populations (children with mixed anxiety disorders (MAD)). First, we hypothesized that children with SAD would show heightened subjective and late ERP reactivity (LPP; [17, 18, 20]) in response to angry faces with socially threatening interpretations, but no differences in early ERPs (P100, N1700, EPN; [16–18]) when compared to HC children. Second, based on previous findings reporting reappraisal difficulties in adult SAD [6], we hypothesized that, compared to HC children, children with SAD would show smaller reappraisal effects on subjective and LPP responses (i.e. smaller reappraisal-related reductions of subjective arousal and LPP amplitudes). Third, we expected reappraisal to be more effective in older children compared to younger children, consistent with the predictions of developmental literature [22, 25]. Since other childhood anxiety disorders (e.g. generalized anxiety disorder, separation anxiety, and specific phobia) have also been characterized by enhanced ERP reactivity in response to threatening faces [20] and reduced reappraisal ability [8], we did not make any predictions for disorder specific outcomes (i.e. difference between SAD and MAD groups). Lastly, we exploratory investigated potential gender differences on subjective and neural reactivity as well as on effects of cognitive reappraisal [35, 36].

## Methods

### Participants

Children were recruited for a three-session research project investigating cognitive and behavioral emotion regulation and emotional reactivity in childhood SAD. The final sample consisted of 85 children aged 10–13 years. Participants were recruited through local schools, newspapers and radio advertisements. All participants and their parents provided informed consent. Participants were paid 100 Euro in age-appropriate vouchers upon study completion. Ethical approval was granted by the local ethics committee.

Inclusion criterion for the SAD group was SAD as a primary diagnosis (*n* = 28). Children in the MAD clinical control group (*n* = 28) met DSM-5 criteria for separation anxiety disorder (*n* = 6), specific phobia (*n* = 11) or generalized anxiety disorder (*n* = 11) as a primary diagnosis, and they did not have a comorbid SAD. HC children (*n* = 29) did not meet criteria for any lifetime mental disorder. All children were Caucasians. Exclusion criteria for all groups were current or past psychotic episodes, severe major depression, pervasive developmental or neurological disorders and previous or current psychotherapeutic treatment. Diagnostic status of eligible children was assessed by trained graduate students using a modified version of the Anxiety Disorders Interview Schedule for children (Kinder-DIPS; [37]). Parents and children were interviewed separately. All diagnostic sessions were videotaped and supervised by a licensed clinical psychologist. Children also completed the short version of the culture fair intelligence test (CFT-20; [38]).

### Psychometric Measures

Children filled out the Social Phobia and Anxiety Inventory for Children (SPAI-C; [39]), a self-report questionnaire that assesses social fears, and the Fear Survey Schedule for Children (FSSCR; [40]), a self-report measure for different phobic fears. Additionally, children self-reported cognitive, affective and behavioral symptoms of depression by filling out the German version of the Children’s Depression Inventory (CDI; [41]). Parents filled out the Child Behavior Checklist (CBCL; [42]), which measures parent’s reports of emotional and behavioral problems. Internal consistency for all psychometric measures in the present sample was good to excellent (α = 0.84–0.96).

### Procedure and Stimuli

Stimuli were obtained from the Radboud Faces Database (RaFD; [43]) and consisted of 8 Caucasian children (4 boys and 4 girls) with angry facial expressions. Contrary to other studies in the field (e.g. [16]), only pictures of children displaying anger were used. This was done since anger may trigger specifically fear of negative evaluation (e. g. “He is angry with me because I did something wrong”), which is the most relevant emotion leading to enhanced reactivity in SAD children. All stimuli (1680 × 1050 pixels) were displayed in the center of a 22″ computer screen using Presentation software (Neurobehavioral Systems, Albany, California). Stimuli were presented in two experimental conditions. In the *reactivity condition*, four different pictures of children with angry facial expressions were displayed, and participants were instructed to appraise the stimulus as socially threatening (e.g. “Tom looks angry because he doesn’t like you”). In the *reappraisal condition*, another four different angry children’s faces were shown, and participants were instructed to reappraise the face to make the stimulus less socially threatening (e.g. “Max looks angry because of a bad grade. This has nothing to do with you”).

**Fig. 1:**
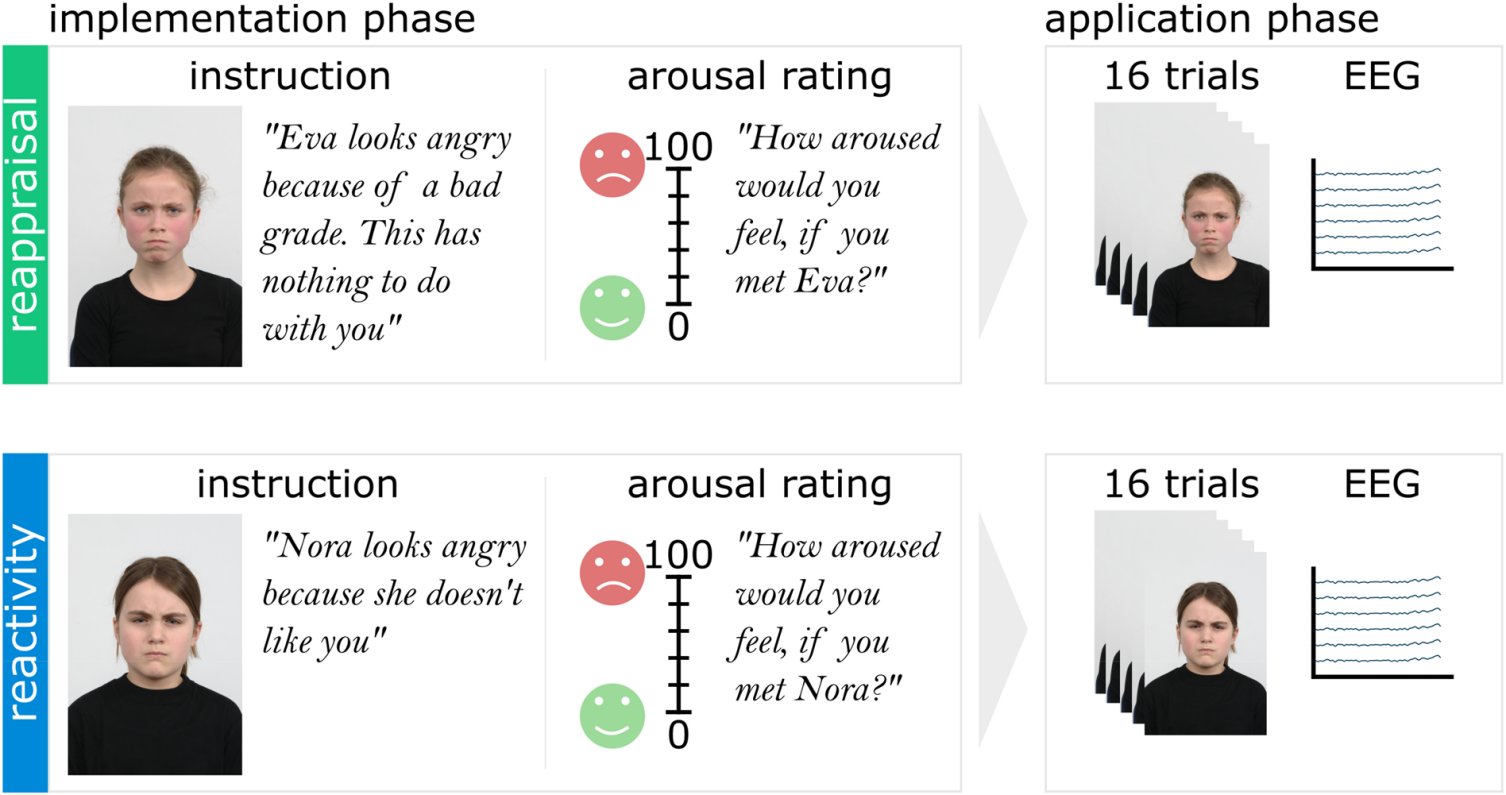
Reappraisal task. Each block of trials (three blocks per condition) consisted of an implementation phase followed by an application phase. In the implementation phase, children viewed four different angry children’s faces and implemented the instructions given for that condition. After each children’s face was presented, children rated how aroused they would feel if they were to meet the presented child. In the application phase, the four children’s faces from the implementation phase were presented four times each in randomized order, and children continuously applied the instructions from the implementation phase

The experiment started with a reappraisal training, during which children were introduced to the concept of cognitive appraisal. To ensure that children understood the idea of cognitive appraisal, children had to generate two examples of their own: one where another child looked angry, but this had nothing to with them (reappraisal) and another example where another child looked angry because this child was mad at them (reactivity). Participants then practiced the appraisal task for two trials and rated their arousal (cf. [22]). Following, participants started the 6-block (3 reactivity, 3 reappraisal) task. The conditions were alternated and the condition type in the first block was counterbalanced across participants. Each block consisted of an *implementation phase* and an *application phase* (see Fig. 1; cf. [44]). During the self-paced implementation phase participants viewed four angry children’s faces and applied the instruction given for that condition (reappraisal vs. reactivity). Then, participants rated how aroused they would feel if they met each child on a visual analogue scale (0 = not aroused to 100 = very aroused).

During the application phase, the same four actors shown in that block’s implementation phase were presented four times each for 2000 ms in randomized order (intertrial intervals ranged from 800 to 1200 ms) with the restriction of no more than three repetitions of the same stimulus in a row. This resulted in 16 trials per each block and 48 trials per condition. The assignment of stimulus to condition was counterbalanced across participants, thus stimuli in the reappraisal condition were not shown in the reactivity condition and vice versa. As a manipulation check, children evaluated how well they were able to implement the instructions on a 5-point scale (0 = not well to 4 = very well).^1^ To limit circadian influences, all EEG assessments started between 1:00 and 4:30 pm.

### EEG Recording and Data Reduction

EEG was recorded from 64 active electrodes of the actiCAP system relative to average reference using a QuickAmp 72 amplifier (Brain Products GmbH, Gilching, Germany) at a continuous rate of 500 Hz. Electrodes were positioned following the 10–20-system. Four electrodes were used to record the electrooculogram (EOG) from positions below and above one eye and from the outer canthi of both eyes. Impedances were kept below 20 kΩ [45–47].

Offline data processing was performed with EEGLAB [48] and Matlab software. All scalp channels were re-referenced to mathematically linked mastoids. Problematic channels were removed using EEGLAB automatic channel rejection algorithms (EEGLAB function rejchan; spectrum criteria in a range 1–250 Hz on normalized data with 5 SD threshold). Independent Component Analysis (ICA) was used to computationally remove ocular artifacts. An Infomax algorithm was trained on 4 s (− 1000 to 3000 ms) epochs of 1 Hz high-pass filtered data after removing noisy epochs using EEGLAB autorej function. SASICA, an EEGLAB toolbox plug-in, was used for semi-automatic detection of independent components representing ocular artifacts [49] using the ADJUST and EOG correlation methods. The components identified as artifacts by SASICA were individually reviewed and then removed before reconstructing the full length of unfiltered continuous data. The ICA-pruned data was band-pass filtered from 0.1 to 30 Hz and cut into segments covering − 200 to 2000 ms from stimulus onset (pre-stimulus voltage removed as baseline). Artifacts were identified using the threshold criterion (± 125 µV). One participant was excluded from analysis due to loss of more than 50% of trials from one experimental condition. In the final sample, on average 90.72% (*SD* = 10.42%; range 51–100%) of the epochs were retained. This resulted in at least 23 (on average 43.55) single trials per each mean analyzed in this study, exceeding the recommendation of 8 trials sufficient for obtaining reliable ERP estimates [50]. The epoch retention rate was independent of condition, participant gender, age and group (*p*s > 0.098; repeated measures ANOVA). Following [51], we computed Cronbach’s alpha estimates for all the ERP components analyzed in this study by treating the average amplitudes for the two conditions as items. Internal consistencies were good to excellent (alpha = 0.81–0.97) and above the recommended ERP reliability threshold (alpha = 0.70; [52]).

**Fig. 2:**
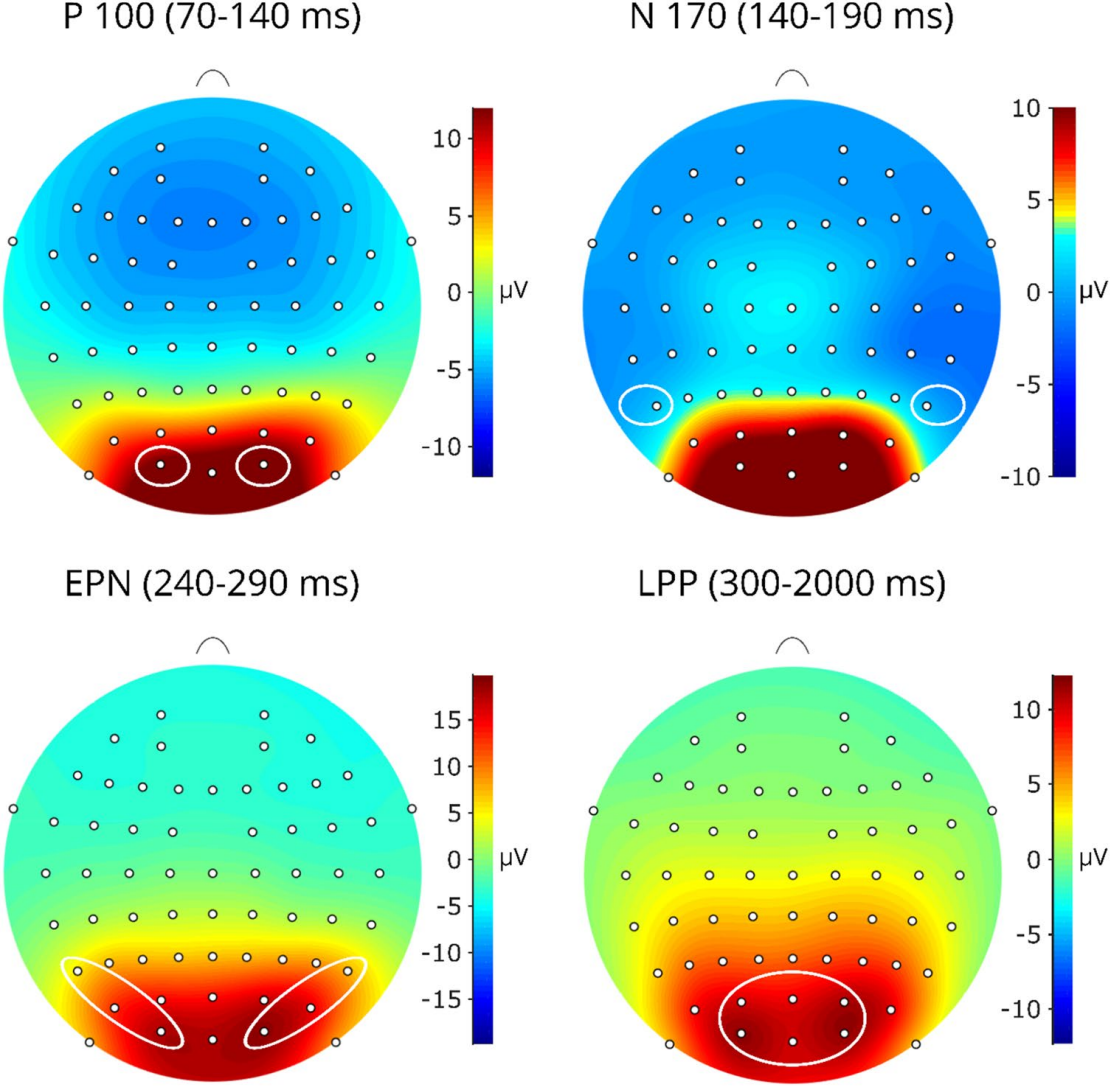
Scalp distribution of ERP amplitudes averaged across participants and condition. Circles indicate the electrode clusters used to quantify the ERPs

ERPs were assessed by separately averaging trials for each condition (reactivity, reappraisal). ERP components were quantified as the mean amplitude (µV) in the following electrode positions and time windows, chosen according to previous studies and visual inspection. P100 at O1, O2 (70–140 ms; [53, 54]). N170 at P7, P8 (140–190 ms; [55, 56]), and EPN at P7, P8, PO7, PO8, O1, and O2 (240–280; [44, 55]). The LPP was quantified as the mean amplitude at O1, O2, Oz, PO3, PO4 and POz, based on visual inspection of the scalp distribution (see Fig. 2) and previous LPP studies with children [20, 56–58]. In accordance with previous child studies (cf. [14, 20, 22, 59]), we analyzed the LPP in three separate time windows at 300–600 ms (early window), 600–1000 ms (middle window) and 1000–2000 ms (late window).

### Statistical Analyses

Children were divided into younger (*n* = 44; mean age 10.9 years, *SD* 0.5 years) and older (*n* = 41; mean age 12.7 years, *SD* 0.5 years) groups based on a median split (cf. [21]). Mixed-design ANOVAs were computed to evaluate the effects of each Condition (reactivity, reappraisal) as within-subjects factors and Gender (boys, girls), Age (young, old) and Group (SAD, MAD, HC) as between-subjects factors on subjective and neural outcomes (P100, N170, EPN, LPP). For the LPP, we also included Time (300–600 ms, 600–1000 ms, and 1000–2000 ms) as a within-subjects factor in the analysis. When appropriate Greenhouse–Geisser corrections were applied, in which case corrected *p*-values and degrees of freedom were reported. For the sake of brevity, we report follow-up tests only in case of significant or marginally significant effects involving Group, Age or Gender. In case of significant main effects involving Group, we used post hoc Tukey tests. Simple effects analyses were used for follow-up testing of significant interaction effects. Measures of effect size for ANOVAs are expressed as η_p_^2^.

## Results

### Participant Characteristics

Sample characteristics are summarized in Table 1. Groups did not significantly differ in age or cognitive abilities (*p*s > 0.616). As expected, children with SAD had significantly higher scores in social anxiety symptom measures (SPAI-C) than children in HC and MAD groups. The mean SPAI-C score in the SAD group was above the clinical cut-off (20; [60]).

**Table 1:** Sample characteristics

	SAD (*n* = 28)		MAD (*n* = 28)		HC (*n* = 29)		Statistics	
	*n*	*%*	*n*	*%*	*n*	*%*	χ^2^ (*df* = 2)	*p*
Gender (female)	22	78.57	13	46,43	11	37.93	10.47	0.005

	*M*	*SD*	*M*	*SD*	*M*	*SD*	*F*(2,82)	Post-Hoc Tukey	*d* (95% CI)
Age (years)	11.81	1.17	11.93	1.00	11.69	0.83	0.40; n.s	.	
CFT-20	112.00	11.40	108.75	14.54	109.90	11.36	0.49; n.s	.	
FSSCR	62.79	19.55	47.14	17.29	26.52	18.30	27.89**	SAD > HC**	1.92 (1.23–2.54)
								MAD > HC**	1.16 (0.60–1.72)
								SAD > MAD**	0.85 (0.30–1.40)
SPAI-C	21.61	5.95	6.24	4.39	4.84	4.27	100.71**	SAD > HC**	3.25 (2.46–4.04)
								SAD > MAD**	2.94 (2.18–3.70)
CDI	12.32	6.86	6.89	3.14	5.34	3.98	15.70**	SAD > HC**	1.25 (0.68–1.82)
								SAD > MAD**	1.02 (0.46–1.58)
CBCL Total	25.29	12.81	22.36	15.39	11.44	10.99	8.39**	SAD > HC**	1.16 (0.60–1.72)
								MAD > HC*	0.82 (0.23–1.36)

*MAD* Mixed Anxiety Disorders including separation anxiety disorder, specific phobia and generalized anxiety disorder, *SAD* Social Anxiety Disorder, *CFT-20* Intelligence Test Short Version, *FSSCR* Fear Survey Schedule for Children, *SPAI-C* Social phobia and anxiety inventory for children, *CDI* Children’s Depression Inventory, *CBCL* Child Behavior Checklist

**p < .01;*p < .05

### Subjective Arousal

**Fig. 3:**
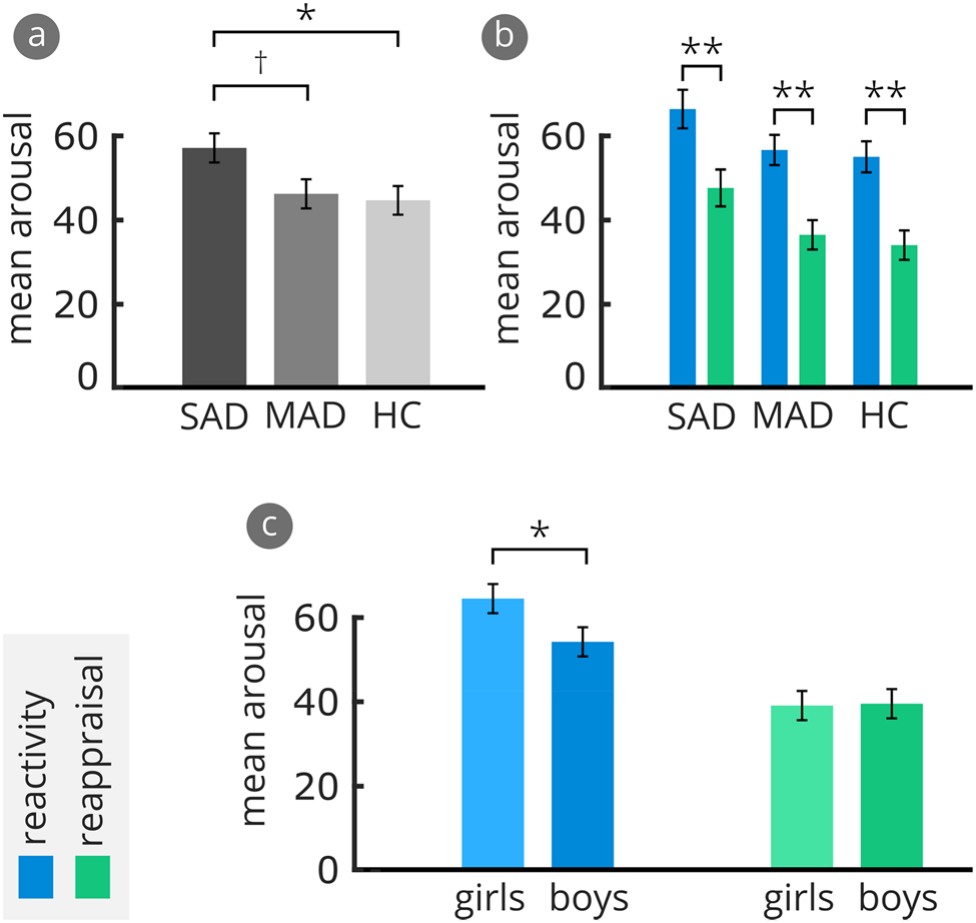
Subjective Arousal. **a** Mean arousal to angry children’s faces averaged across both conditions separately for each group, **b** mean arousal separately for the two conditions for each group, **c** mean arousal separately for the two conditions and for boys and girls. Error bars show standard errors

The 3 (Group: SAD, MAD, HC) X 2 (Age: young, old) X 2 (Gender: boys, girls) mixed-design ANOVA showed a marginally significant main effect for Group, *F*(2,73) = 2.55, *p* = 0.085, η_p_^2^ = 0.065: SAD children rated angry children’s faces as more arousing in both conditions than HC, *p* = 0.027, *d* = 0.63, and also marginally more arousing than MAD children, *p* = 0.055, *d* = 0.54, while MAD and HC did not differ from each other, *p* = 0.960 (see Fig. 3a). Generally, all children rated the faces in the reactivity condition as more arousing than the faces in the reappraisal condition (see Fig. 3b), as indicated by a significant main effect, *F*(1,73) = 73.58, *p* < 0.001, η_p_^2^ = 0.502. As indicated in Fig. 3c, girls rated the faces in the reactivity condition as more arousing than boys, *p* = 0.044, *d* = 0.68 (no gender differences in the reappraisal condition, *p* = 0.928), as indicated by a significant interaction between Gender and Condition, *F*(1,73) = 5.24, *p* = 0.025, η_p_^2^ = 0.067.

### Early ERPs (P100, N170, EPN)

For each ERP component a 3 (Group: SAD, MAD, HC) X 2 (Age: young, old) X 2 (Gender: boys, girls) mixed-design ANOVA was run. Early ERPs did not differ by Group or Gender, *Fs* < 2.57, *ps* > 0.114. There was a significant main effect for Age on the P100, *F*(1,73) = 9.49, *p* = 0.003, η_p_^2^ = 0.115, reflecting higher ERP amplitudes in younger children compared to older children. Similarly, younger children showed higher EPN-amplitudes than older children, *F*(1,73) = 4.89, *p* = 0.030, η_p_^2^ = 0.063 (see Table 2).

**Table 2:** Means and standard deviations of ERP amplitudes in younger and older children

	Younger children (*n* = 44)		Older children (*n* = 41)	
	*M*	*SD*	*M*	*SD*
P100	15.89	6.84	11.32	6.25
EPN	13.55	4.76	11.24	5.30
LPP	13.23	4.98	10.51	4.49

### Late ERPs (LPP in Three Time Windows: 300–600; 600–1000; 1000–2000 ms)

**Fig. 4:**
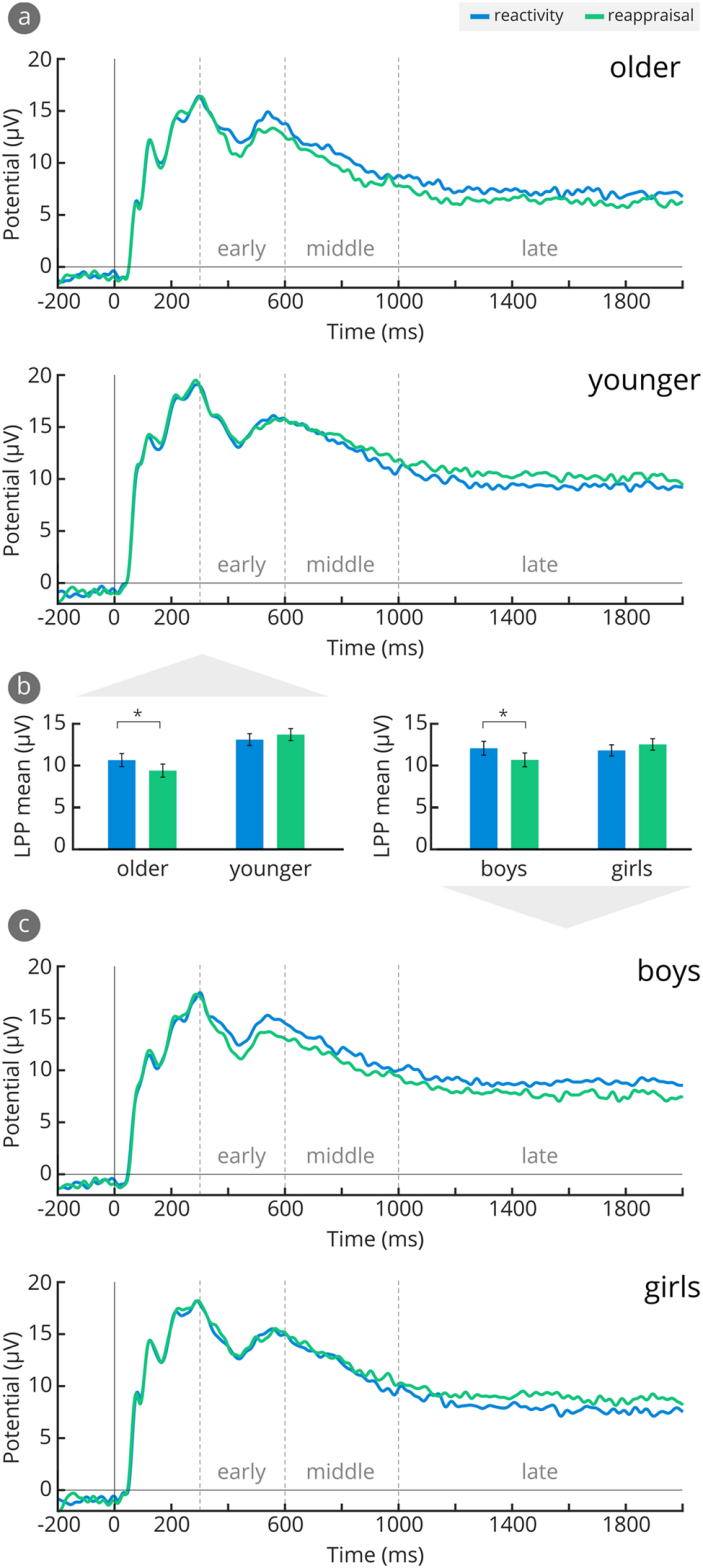
Cognitive appraisal related effects on LPP (averaged from Oz, O1, O2, POz, PO3 and PO4). **a** Stimulus-locked ERPs elicited by socially threatening vs. reappraised angry children’s faces across older vs. younger children, **b** mean LPP amplitudes for socially threatening vs. reappraised angry children’s faces across older vs. younger children, and boys vs. girls, **c** stimulus-locked ERPs elicited by socially threatening vs. reappraised angry children’s faces across boys vs. girls. Error bars show standard errors

The 3 (Group: SAD, MAD, HC) X 2 (Age: young, old) X 2 (Gender: boys, girls) X 3 (Time: 300–600 ms, 600–1000 ms, and 1000–2000 ms) mixed-design ANOVA with repeated measures on Time showed no significant effects involving Group, on LPP amplitudes *Fs* < 2.79, *ps* > 0.068. LPP amplitudes differed according to Time, *F*(1.43, 104.42) = 101.85, *p* < 0.001, η_p_^2^ = 0.582, and Age, *F*(1,73) = 10.48, *p* = 0.002, η_p_^2^ = 0.126. Similar to early ERPs, younger children showed higher LPP amplitudes than older children (see Table 2). Furthermore, Condition interacted with Age, *F*(1,73) = 6.22, *p* = 0.015, η_p_^2^ = 0.079 and Gender, *F*(1,73) = 7.94, *p* = 0.006, η_p_^2^ = 0.098: Older children showed lower LPP amplitudes in the reappraisal condition compared to the reactivity condition, as expected, *p* = 0.025, *d* = 0.26. Younger children, by contrast, did not show these differences, *p* = 0.238. The Condition X Gender interaction was due to reappraisal effects in boys (irrespective of age), with lower LPP amplitudes in the reappraisal than in the reactivity condition, *p* = 0.019, *d* = 0.38, but not in girls, *p* = 0.135 (see Fig. 4b). The ERP waveforms in Fig. 4a and c illustrate these two interactions.

## Discussion

The present study is the first to investigate the differences in subjective and cortical responses in children with SAD, healthy controls (HC) and a clinical control group with mixed anxiety disorders (MAD) by means of cognitive appraisals (reactivity, reappraisal). Importantly, all children showed reduced subjective reactivity following reappraisal, supporting cognitive reappraisal as a very beneficial emotion regulation strategy already in children with anxiety disorders. Neurally, a reduced electrocortical reactivity following reappraisal was evident only in older children and in boys. In accordance with our hypothesis, this suggests that the LPP provides a measure of emotion regulation, especially in older pediatric populations. Furthermore, we predicted that, compared to HC children, children with SAD would show increased subjective and late ERP responses to socially threatening children’s faces. This hypothesis was partly confirmed, children with SAD showed (a) generally heightened subjective reactivity to angry children’s faces in both conditions (reactivity and reappraisal), but (b) no differences in ERPs when compared to HC children. Finally, contrary to our hypothesis, we did not find any differences between SAD, MAD or HC groups in reappraisal-related changes of subjective or LPP outcome measures.

### Enhanced Subjective Reactivity in SAD Children, But No Clinical Effects on ERP Reactivity or Reappraisal Modulation

The results showed that children with SAD responded with heightened subjective reactivity in both conditions when compared to HC and MAD. An overall enhanced subjective reactivity in SAD children indicates that SAD children evaluate an angry looking peer as threatening and that this cannot be fully reduced by means of cognitive appraisal as applied in our brief experimental intervention. Still, cognitive reappraisal had a positive effect on subjective arousal across all participants, including both clinical groups, underpinning its value for addressing negative emotion in childhood anxiety disorders.

Interestingly, the enhanced subjective reactivity in SAD children was not reflected in their ERPs. Such discordance between subjective and neural responses has been seen before, in both children [14] and adults [61]. In addition to the absence of group differences in ERP reactivity, groups did not differ in subjective or electrocortical response modulation using reappraisal. As expected, compared to the reactivity condition, all children reported lower subjective arousal in the reappraisal condition. Yet, this did not translate uniformly to the neural indices since a reappraisal-related LPP reduction was only present in older children and in boys. Generally, the (dys-)coherence between different emotional subsystems (physiological, experiential, behavioral) can vary by interindividual differences, experimental setups, the type of assessed emotion, psychopathology and by developmental stages of participants [61, 62]. In this line, previous research suggests that neural correlates of emotion (dys-)regulation in anxiety most likely develop at older ages during late childhood and adolescents, leading to discordance between subjective and neural measures in younger samples [14, 25], presumably due to brain maturation and increased cognitive control in older children [62]. Taken together, this lack of coherence between subjective and electrocortical measures emphasizes the importance of assessing emotional reactivity and regulation with multiple methods [63] and in samples with a broad age range of children and adolescents to capture potential developmental changes in emotional coherence.

Our study partially replicates, and importantly extends, previous research findings using children’s faces as stimuli. Several studies similarly did not find differences between children with SAD and HC in both early [16–18] and late [16] ERP responses to emotional adult faces, while other studies have reported enhanced LPPs in response to threatening adult faces [17, 18, 20]. A previous study found no group differences in habitual use of reappraisal in children with SAD when compared to HC [64, 65], which confirms our clinical null findings. However, the absence of group differences in the effectiveness of reappraisal conflicts with existing studies, which reported reappraisal deficits in older children aged 10–17 with different anxiety disorders, including SAD [7–9]. They also contrast with adult findings suggesting greater reappraisal-related brain activation in controls compared to adults with SAD [66].

The absence of group differences in ERP reactivity and reappraisal modulation might be explained by changes in cognitive and neural development. Reappraisal-related neural activity may be influenced by brain maturation and associated increased cognitive control, working memory and cognitive flexibility [23, 67]. Anatomically, emotion regulation is associated with activity in the prefrontal cortex [68], which is one of the last brain structures to develop in adolescence [69]. In the present study, the reappraisal-related LPP activity showed the expected direction only in the older group, which demonstrates that older children are able to accomplish this non-trivial cognitive operation. In line with this finding, several studies did not find reappraisal-induced LPP reductions in younger children aged 5–12 years [22, 25, 56, 59, 70]. In considering these developmental aspects, it is conceivable that all young children, regardless of their level of anxiety, (a) display bottom-up driven biases towards threatening information [71] and (b) do not show reappraisal modulation in neural components. With increasing age, non-anxious children might learn to inhibit automatic responding to threat by using top-down control strategies, whereas anxious children fail to do so [71, 72]. This might result in group differences in emotional reactivity and regulation with increasing age. Indeed, a recent meta-analysis [72] has found biased processing of threatening stimuli (i.e. enhanced emotional reactivity) in anxious children becomes more evident as children age. This is in accordance with another ERP study which found decreased LPP amplitudes in response to emotional faces with increasing age in controls, but not in SAD children [16]. Possibly, clinical differences in emotional reactivity and reappraisal would only emerge in an adolescent or adult sample.

### Reappraising Threatening Faces in Children: Gender Matters

We found gender effects on reappraisal and reactivity in both subjective and ERP responses. Compared to boys, girls showed higher subjective reactivity when interpreting angry children’s faces as socially threatening. Additionally, reappraisal-induced LPP reductions were present in boys but not in girls. Gender-related differences in emotional reactivity and regulation have been reported from previous studies with adult populations (e.g. [73–76]. For example, in a study by McRae et al. [35] men showed lower activity in brain regions related to the use of reappraisal such as the prefrontal cortex when compared to woman. On a subjective level, men and woman were equally able to reduce negative affect towards negative pictures through reappraisal. The authors speculate that male participants may require less cortical effort for the regulation of negative emotion, which would correspond to the findings of our study that only boys showed reappraisal effects on neural activity. Furthermore, girls have often been found to report higher reactivity to negative stimuli and greater difficulties regulating their negative emotions [77–79]. These gender-related differences converge with higher prevalence rates of anxiety and mood disorders in girls [80] suggesting a vulnerability for negative emotions and a difficulty to regulate these. There are also studies reporting gender differences on ERP measures: Girls showed enhanced LPPs to unpleasant and neutral versus pleasant stimuli while boys showed enhanced LPPs to pleasant versus neutral stimuli [81]. Another ERP study showed reduced reappraisal effects on the LPP in younger girls [21]. However, the underlying mechanisms of gender effects in children are still unknown and more research on this topic is clearly needed [82]. A better understanding of gender-related differences in emotion regulation is crucial for informing theoretical models [83], and therapeutic interventions, e.g. by addressing emotion regulation strategies specifically for boys versus girls [77].

### Limitations and Future Research

Our sample size did not allow for specific comparisons between single anxiety disorders (e.g. specific phobia vs. generalized anxiety disorder), which should be further investigated in future studies. Additionally, to evaluate age and gender effects in more detail, future research should include larger samples of girls and boys across a broad age range. Further, since we did not include a neutral baseline condition (e.g. observe, cf. [44]), we do not know whether the subjective hyperreactivity in SAD was due to a generalized reactivity to any kind of social information or due to reporting biases. Last, we cannot rule out that children with SAD reported an elevated subjective reactivity in both conditions of our experiment due to a generally elevated emotional state [84]. This would mean that also neutral and non-social stimuli could have led to reports of enhanced arousal (e.g. “How aroused would you feel if you were alone in your own room at home”). Future studies may therefore want to control for such effects using non-social control stimuli and situations.

In conclusion, the present findings indicate that children with SAD report enhanced subjective reactivity in response to angry children’s faces. It appears that LPP modulation can serve as a neural marker of cognitive reappraisal in older children and in boys, regardless of clinical anxiety. Importantly, while previous pediatric SAD research used adult faces, the present study extends previous findings to more salient peer stimuli. The absence of group differences in reappraisal suggests SAD children were just as effective at regulating as HC and MAD and that changing the affective meaning of social situations is something that patients and controls can accomplish. Thus, rather than addressing putative reappraisal deficits, therapeutic interventions in children with (social) anxiety disorders should target generalized hyperreactivity to social cues.

## Summary

This is the first study known to us which experimentally investigated subjective and neural reactivity to threatening children’s faces along effects of cognitive reappraisal in a sample of children with SAD, mixed anxiety disorders and healthy controls. Importantly, our results provide evidence, that children with SAD may not only show an enhanced subjective reactivity to social threatening stimuli but that cognitive reappraisal might be an adequate cognitive strategy to counteract dysfunctional threat processing in children. In line with previous studies, no neural correlates of threat processing in clinical anxiety were found in this preadolescent age group. Interestingly, reduced electrocortical reactivity after reappraisal was further only evident in older children and boys and was again unrelated to anxiety. Together with previous research our study therefore suggests that neural correlates of anxiety related threat processing may emerge at later ages of development and that both age and gender of participants may play important influencing factors in this field of research.

## Footnotes

1 A univariate analysis of variance (ANOVA) with child gender, age and group as independent variables and children’s rating on how well they were able to implement the instruction as dependent variable did not yield any significant effects (*ps* > 0.152; *F*s < 2.10).
